# C5aR2 Deficiency Lessens C5aR1 Distribution and Expression in Neutrophils and Macrophages

**DOI:** 10.1155/2024/2899154

**Published:** 2024-07-10

**Authors:** Ting Zhang, Ning Ma, Jiaxing Wang, Xiaoyun Min, Linlin Wei, Ke Li

**Affiliations:** ^1^ Department of Pathology The Second Affiliated Hospital School of Medicine Xi'an Jiaotong University, Xi'an 710004, China; ^2^ Core Research Laboratory The Second Affiliated Hospital School of Medicine Xi'an Jiaotong University, Xi'an 710004, China; ^3^ Institute of Hematology School of Medicine Northwest University, Xi'an 710069, China

## Abstract

As another receptor for complement activation product C5a, C5aR2 has been paid much attention these years. Although controversial and complex, its specific signals or roles in modulating the classic receptor C5aR1 have been investigated and gradually revealed. The hypothesis of the heterodimer of C5aR1 and C5aR2 has also been suggested and observed under extremely high C5a concentrations. In this article, we tried to investigate whether C5aR2 would affect C5aR1 expression under normal or inflammatory conditions in WT and *C5ar2*^*-/*-^ mice of C57BL/6 background. We focused on the innate immune cells—neutrophils and macrophages. The mRNA levels of *C5ar1* in normal kidney, liver, and the mRNA or protein levels of naïve-bone marrow and peripheral blood leukocytes and peritoneal M*φ*s were comparable between WT and *C5ar2*^*-/*-^ mice, indicating the technique of C5aR2 knockout did not affect the transcription of its neighboring gene C5aR1. However, the mean fluorescence intensity of surface C5aR1 on naïve circulating *C5ar2*^*-/*-^ neutrophils detected by FACS was reduced, which might be due to the reduced internalization of C5aR1 on *C5ar2*^*-/*-^ neutrophils. In the peritonitis model induced by *i.p*. injection of thioglycollate, more neutrophils were raised after 10 hr in *C5ar2*^*-/*-^ peritoneal cavity, indicating the antagonism of C5aR2 on C5aR1 signal in neutrophil chemotaxis. After 3 days of thioglycollate injection, the mainly infiltrating macrophages were comparable between WT and *C5ar2*^*-/*-^ mice, but the *C5ar1* mRNA and surface or total C5aR1 protein expression were both reduced in *C5ar2*^*-/*-^ macrophages, combined with our previous study of reduced chemokines and cytokines expression in *C5ar2*^*-/*-^ peritoneal macrophages, indicating that C5aR2 in macrophages may cooperate with C5aR1 inflammatory signals. Our article found C5aR2 deficiency lessened C5aR1 distribution and expression in neutrophils and macrophages with different functions, indicating C5aR2 might function differently in different cells.

## 1. Introduction

Complement, as a finely tuned ancient innate immune system, functions mainly as the first-line defender against injuries and infections [1]. It is composed of about 50 proteins, including complement components, regulatory proteins, and receptors. Three complement activation pathways converge at the cleavage of C3, leading to the release of small fragment C3a and downstream proteolytic product C5a, which both have important physiological and pathological functions. These small fragments are called anaphylatoxins, exerting their unique functions through specific interactions with their receptors respectively [2].

Different from C3a and its receptor C3aR, there are two receptors for C5a, C5aR1 and C5aR2, the latter also known as GPR77 or C5L2 [3]. C5aR1, as the firstly identified receptor for C5a in 1991, its mainly role in inducing overwhelming inflammation upon binding with C5a has been well defined. It is widely expressed on myeloid (*e.g*., neutrophils, monocytes/macrophages (Mo/M*φ*s)) as well as parenchymal cells (e.g., epithelium, endothelium). C5aR1 and C3aR both are the classical seven transmembrane G-protein coupled receptors (GPCRs) [2]. C5aR2, found in 2000, is structurally homologous with C5aR1 [4]. Similar to C5aR1, it can bind to C5a and the terminal arginine degradation form des Arg C5a. C5aR2 is expressed alongside C5aR1 and usually at lower levels. Although being one member of the GPCRs superfamily, due to changes in certain critical transmembrane and intracellular motifs, C5aR2 is a nonclassical GPCR, following ligand binding, it is unable to couple G-proteins and downstream GPCR signals [5, 6].

Thus, it is firstly considered as a decoy or scavenger receptor for C5a, antagonizing C5a-C5aR1 signaling, supported by several studies [5, 6, 7]. But others have showed that instead of the G-proteins, the possibility that other signaling pathways mediated by C5a-C5aR2 may exist, e.g., *β*-arrestins [8, 9]. Depending on disease models studied, or even in the same disease studied by different groups or from different perspectives, C5aR2 functions either as pro- and/or anti-inflammatory mediators, such as in sepsis [10, 11, 12] and allergic asthma [13].

The controversial role of C5aR2 in diseases and its complex interaction with C5aR1 make it enigmatic. Heterodimerization or intracellular interaction between C5aR1 and C5aR2 has been proposed as regulatory mechanisms for C5aR2 function. Using immunofluorescence co-localization staining and bioluminescence resonance energy transfer measurement, Bamberg and Croker's studies support the antagonistic role of C5aR2 to C5aR1, respectively, that is, inhibiting chemotaxis and ERK1/2 signaling of human neutrophils, or promoting the release of anti-inflammatory factors IL-10 and G-GSF from human monocyte-derived macrophages at significantly high levels of C5a (100–500 nM) [9, 14, 15]. But Hsu et al. found the pro-inflammatory roles of C5aR1 and C5aR2 in a murine model of acute experimental colitis. In this article, they found C5aR2 is required for C5aR1 internalization and ERK signaling in mouse bone marrow-derived macrophages by co-immunoprecipitation and fluorescence resonance energy transfer [16]. It seems that in neutrophils and macrophages, C5aR2 may function differently to coordinate C5aR1. This dual nature of C5aR2 and its relationship with C5aR1 needs more investigation on specific cellular or tissue environment [13, 17].

In our previous study, using a mouse model of ascending urinary tract infection, we have demonstrated the comparable pro-inflammatory and pathogenic roles of C5aR1 and C5aR2 in acute pyelonephritis with different specific mechanisms, that is, C5a/C5aR1 axis contributes to renal inflammation and tissue damage through mediating excessive inflammation and possibly providing potential binding site for *Escherichia coli* on renal tubular epithelial cells (RTECs), while C5a/C5aR2 axis through upregulation of HMGB1 and NLRP3 inflammasome in M*φ*s [18, 19]. In the present study, using C5aR2 deficient mice (*C5ar2*^*-/*-^), we aimed to evaluate the C5aR1 expression in/on myeloid neutrophils and Mo/M*φ*s from WT and *C5aR2*^*-/*-^ mice, trying to assess whether C5aR2 knockout would affect the distribution and expression of C5aR1 as one way of the regulation or interaction between these two receptors.

## 2. Materials and Methods

### 2.1. Materials

We used the following reagents and materials: monoclonal rat anti-mouse CD45 (30-F11, PE-Cy7), Ly6G (1A8, Percp), CD11b (M1/70, APC), C5aR1 (20/70, PE), and purified rat IgG2b isotype control (RTK4530, PE) (all BioLegend, USA); polyclonal rat anti-mouse C5aR2 (Hycult, Netherlands); cell culture medium DMEM-F12 and RPMI-1640, FCS, insulin-transferrin-selenium solution, penicillin, streptomycin, BSA, Fast SYBR® Green Master Mix (all Thermo Fisher, USA); hydrocortisone, tri-iodothyronine and TRIzol (all Sigma-Aldrich, USA); FcR-blocking antibody (CD16/32, 2.4G2), thioglycollate (TG), Cytofix/Cytoperm Fixation/Permeabilization Kit (BD Biosciences, USA); M-MLV, oligo dT, dNTP mix, RNase inhibitor, GoTap DNA polymerase (all Promega, USA); collagenase II (Worthington, USA).

### 2.2. Mice. *C5ar2*^*-/*-^

Mice were kindly provided by Professor Bao Lu from the Pulmonary Division, Department of Pediatrics, Havard Medical School (Boston, USA). The method for generating these knockout transgenic mice by homologous recombination has been described elsewhere [7]. We backcrossed them onto the C57BL/6 parental strain to generate homozygous *C5ar2*^*-/*-^mice and their wildtype (WT) littermates. Gene and protein depletion of *C5ar2*^*-/*-^ were provided (Figure S1). Mice were bred and maintained in the Animal Center of Xi'an Jiaotong University-specific pathogen-free (SPF) facility, and male or female mice at 8–10 week of age were used. All animal procedures and experiments were approved and oversaw by the Ethics Review Committee for Animal Experimentation in Xi'an Jiaotong University for animal care and welfare.

### 2.3. Primary Renal Tubular Epithelial Cell Cultures

Isolation of mouse primary RTECs was performed as previously described [18]. Briefly, kidneys were harvested from naïve male mice. Minced cortex and outer medulla were digested with 0.1% collagenase II and passed through a series of sieves to a final sieve of 40 *µ*m. The cells and tubulus were collected and cultured in a DMEM-F12 medium containing 2% FCS, 100 U/mL penicillin, 100 *µ*g/mL streptomycin, 1% insulin-transferrin-selenium, 40 ng/mL hydrocortisone, and tri-iodothyronine (5 × 10^−12^ M). To assess the expression of C5aR1 in RTECs, non-passaged 6 days cultured confluent layers of RTECs derived from WT and *C5ar2*^*-/*-^mice were harvested followed by RT-qPCR.

### 2.4. The Preparation of Leukocytes from Peripheral Blood of WT and *C5ar2*^*-/*-^ Mice

WT and *C5ar2*^*-/*-^naïve mice (males and females) were anesthetized with 0.75% sodium pentobarbital through intraperitoneal (*i.p*.) injection at doses of 200 *μ*L/20 g. The anticoagulant blood was obtained by cardiac puncture with a 2 mL syringe pre-rinsing with heparin. Then, the leukocytes from whole blood were prepared after erythrocyte lysis.

After blood collection, mice were perfused with PBS through their hearts. Then, their kidneys and livers were removed and stored in liquid nitrogen for RNA extraction.

### 2.5. The Preparation of Peritoneal Inflammatory Cells

Inflammatory cells (mix of neutrophils and Mo/M*φ*s or M*φ*s-rich) were prepared from peritoneal lavage of female mice at 10 hr or 3 days after *i.p*. injection of 1 mL of 3% TG. Peritoneal lavage fluid was collected with PBS containing 0.5% BSA.

### 2.6. Assessment of Neutrophils and Mo/M*φ*s Constituent, C5aR1 Expression, and Internalization by FACS

Single-cell suspension of blood leukocytes or peritoneal inflammatory cells was prepared using the method described above. Following erythrocyte lysis, cells were washed and re-suspended in PBS, followed by flow cytometric analysis. The cells were pre-incubated with FcR-blocking antibody (anti-CD16/32) and then stained with rat anti-mouse PE-Cy7-conjugated CD45, Percp-conjugated Ly6G, PE-conjugated C5aR1, and APC-conjugated CD11b antibodies, or the appropriate isotype control antibodies at 4°C for 30 min. In order to detect total C5aR1 (surface and intracellular) expression, we used BD Fixation/Permeabilization Kit to permeabilize cells according to the instructions of the manufacturer. All flow cytometric analysis was performed using Calibur Flow Cytometer (BD Biosciences, Franklin Lakes, NJ, USA) and Flowjo software (Tree Star, USA). For C5aR1 internalization, 4 × 10^5^ cells were incubated with 1–50 nM C5a in RPMI-1640 containing only 1% FCS for 30 min at 37°C. Then, the cells were washed with ice-cold PBS to remove C5a and terminate receptor internalization. And the following staining procedures for FACS were all performed at 4°C. Particularly worth mentioning is that cells from our *C5ar2*^*-/*-^ mice are not suitable for fluorochrome FITC staining. Since the procedure of C5aR2 knockout introduced green fluorescent protein gene, during FACS, the fluorescent dyes with similar emission spectrum with green fluorescent protein, *e.g*., FITC, should be avoided (Figure S2).

### 2.7. Quantitative and Conventional RT-PCR

Total RNA was purified from kidney or liver tissue, RTECs, blood leukocytes, or peritoneal cells from WT and *C5ar2*^*-/*-^mice using TRIzol reagent, followed by cDNA synthesis. The quantitative RT-PCR (RT-qPCR) was performed with Fast SYBR® Green Master Mix on a Step One™ Real-time PCR instrument (Thermo Fisher, USA). The 2^−*ΔΔ*Ct^ method with normalization to *β*-actin and controls was used for calculation. The conventional RT-PCR consisted 35 cycles of 1 min at 94°C, 1 min at 60°C, and 1 min at 72°C. The primers used are listed in Table 1, which were designed by Primer Premier 3.0 according to the principles that primer must span an exon–exon junction or primer pair must be separated by at least one intron on the corresponding genomic DNA.

### 2.8. Statistical Analysis

All data are shown as mean ± SD. Unpaired Student's *t* test or ANOVA was used to determine significant differences between samples. All the analyses were performed using GraphPad Prism 8.0 Software (GraphPad Software, La Jolla, CA, USA). *P* < 0.05 was considered to be significant.

## 3. Results

### 3.1. C5aR2 Deficiency Does Not Affect Total C5aR1 Expression under Naïve Conditions

**Figure 1:**
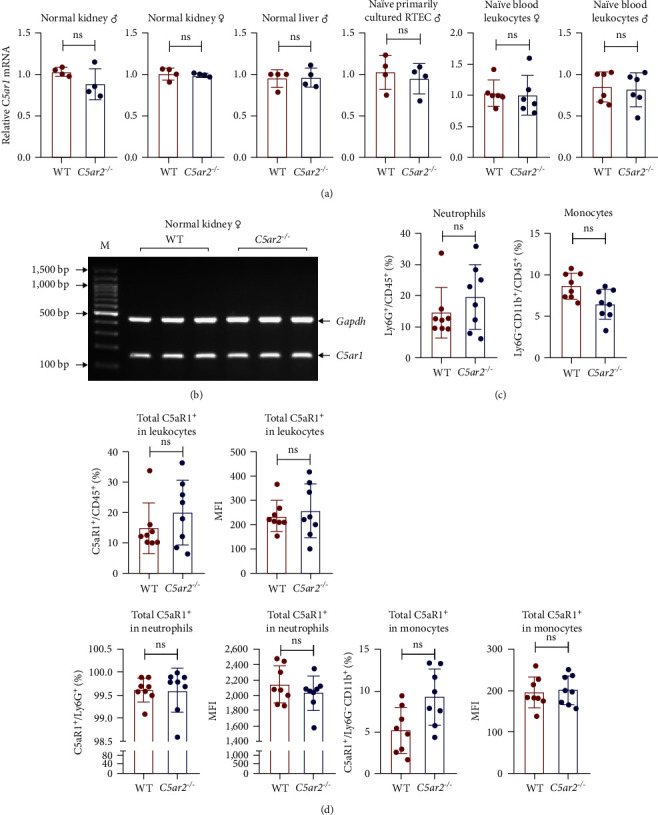
C5aR2 deficiency does not affect total C5aR1 expression under naïve conditions. (a) Relative mRNA levels of *C5ar1* in kidney and liver tissue, naïve primarily cultured RTECs, and peripheral blood leukocytes from normal WT and *C5ar2*^*-/*-^ mice determined by RT-qPCR (*n* = 4/group). (b) A typical agarose gel showing the 155-bp *C5ar1* band and the 453-bp *Gapdh* (internal control) band of conventional RT-PCR of 35 cycles about the *C5ar1* mRNA levels in normal kidneys from female WT and *C5ar2*^*-/*-^ mice. The 100-bp DNA markers (M) are shown alongside the gels. (c) The percentages of neutrophils and monocytes in peripheral blood CD45^+^ leukocytes from normal female WT and *C5ar2*^*-/*-^ mice as assessed by flow cytometry (*n* = 8/group). (d) Total C5aR1 expression in CD45^+^ circulating leukocytes, neutrophils, and monocytes from female WT and *C5ar2*^*-/*-^ mice as assessed by flow cytometry (*n* = 8/group). Each dot represents an individual mouse. ns, no significance, Student's *t* test.

The *C5ar1* mRNA levels in kidney and liver from naïve WT and *C5ar2*^*-/*-^ mice under SPF conditions were compared by conventional RT-PCR, agarose gel electrophoresis, and RT-qPCR. There were no differences in tissue *C5ar1* mRNA levels between these two mice (Figures 1(a) and 1(b)). Then, in order to access the *C5ar1* transcription in cellular level, we chose primarily cultured mouse RTECs and peripheral blood leukocytes (Figure 1(a)). Still, C5aR2 deficiency did not affect *C5ar1* mRNA transcriptions in these two cells. To confirm its expression on protein level, we compared C5aR1 protein levels in WT and *C5ar2*^*-/*-^ peripheral blood leukocytes including its constituent neutrophils and monocytes, as well as bone marrow neutrophils and monocytes by FACS. The gating method and isotype control are shown in Figure S3. Neutrophils were characterized as CD45^+^Ly6G^+^ cells, and monocytes were characterized as CD45^+^Ly6G^−^CD11b^+^ cells. It showed that the percentages of neutrophils and monocytes in peripheral blood and bone marrow CD45^+^ leukocytes from normal WT and *C5ar2*^*-/*-^ mice were comparable (Figure 1(c) and Figure S4(a)) and so did total C5aR1 expressions in peripheral blood leukocytes, peripheral and bone marrow neutrophils and monocytes between WT and *C5ar2*^*-/*-^ mice (Figure 1(d) and Figure S4(b)). Further, there were no differences in C5aR1 expression in peripheral blood neutrophils and monocytes between female and male mice, so female mice were chosen for the following experiments (Figure S5).

Meanwhile, the FACS results indicated that nearly all neutrophils express C5aR1, with C5aR1^+^ percentage more than 98.5% both in WT and *C5ar2*^*-/*-^circulating neutrophils. The percentage of circulating C5aR1^+^ monocytes was about 2.6%–13.4%.

### 3.2. C5aR2 Deficiency Lessens C5aR1 Distribution on Peripheral Blood Neutrophil Surface under Naïve Conditions

**Figure 2:**
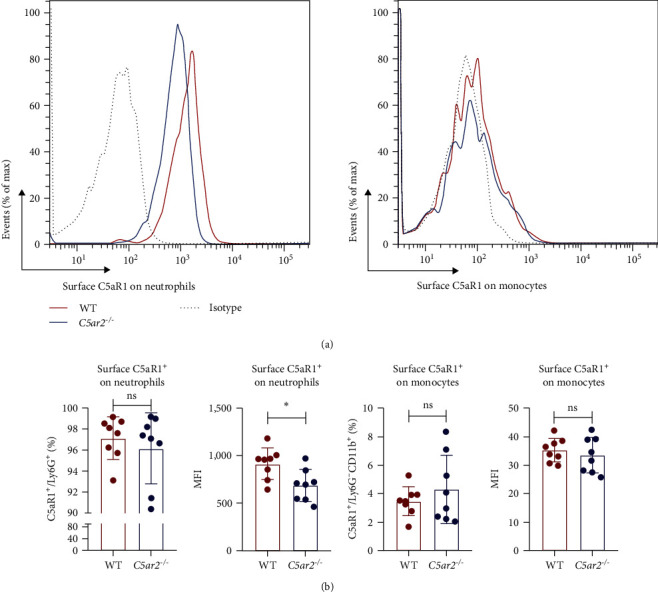
C5aR2 deficiency lessens C5aR1 distribution on peripheral blood neutrophil surface under naïve conditions. (a) Representative FACS analysis of surface C5aR1 expression on peripheral blood neutrophils and monocytes. (b) C5aR1 expression on circulating neutrophils and monocytes from WT and *C5ar2*^*-/*-^ mice as assessed by flow cytometry (*n* = 8/group). Each dot represents an individual mouse.  ^*∗*^*P* < 0.05, ns, no significance, Student's *t* test.

We also accessed the distribution of C5aR1 on neutrophil and monocyte surface of naïve peripheral blood leukocytes by FACS without permeabilization. There were no differences in the percentage of surface C5aR1^+^ neutrophils and monocytes between WT and *C5ar2*^*-/*-^ mice. And the mean fluorescence intensity (MFI) of surface C5aR1 on monocytes was not affected by C5aR2 deficiency. While the MFI of surface C5aR1 on *C5ar2*^*-/*-^neutrophils was less than that on WT neutrophils, which suggested that C5aR2 deficiency may lessen C5aR1 distribution on neutrophil surface under naïve conditions (Figures 2(a) and 2(b)). However, surface C5aR1 distribution was similar between WT and *C5ar2*^*-/*-^ bone marrow neutrophils (Figure S3).

### 3.3. C5aR1 Distribution and Expression Are Similar in Naïve Intraperitoneal M*φ*s between WT and *C5ar2*^*-/*-^ Mice

**Figure 3:**
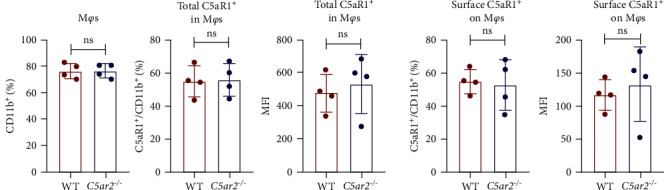
C5aR1 distribution and expression are similar in naïve intraperitoneal M*φ*s between WT and *C5ar2*^*-/*-^ mice. *n* = 8/group. Each dot represents an individual mouse. ns, no significance, Student's *t* test.

As to M*φ*s, we acquired naïve intraperitoneal cells, the percentage of M*φ*s (identified as CD45^+^Ly6G^−^CD11b^+^) in these cells was more than 70% (Figure 3). The C5aR1 distribution and expression were similar in naïve WT and *C5ar2*^*-/*-^ intraperitoneal M*φ*s, as shown in Figure 3 that there were no differences in the percentage of C5aR1^+^ and MFI in both permeabilized and non-permeabilized M*φ*s from these two mice.

### 3.4. Neutrophil Infiltration Is Higher in *C5ar2*^*-/*-^ Mice after 10 hr of 3% TG Injection

**Figure 4:**
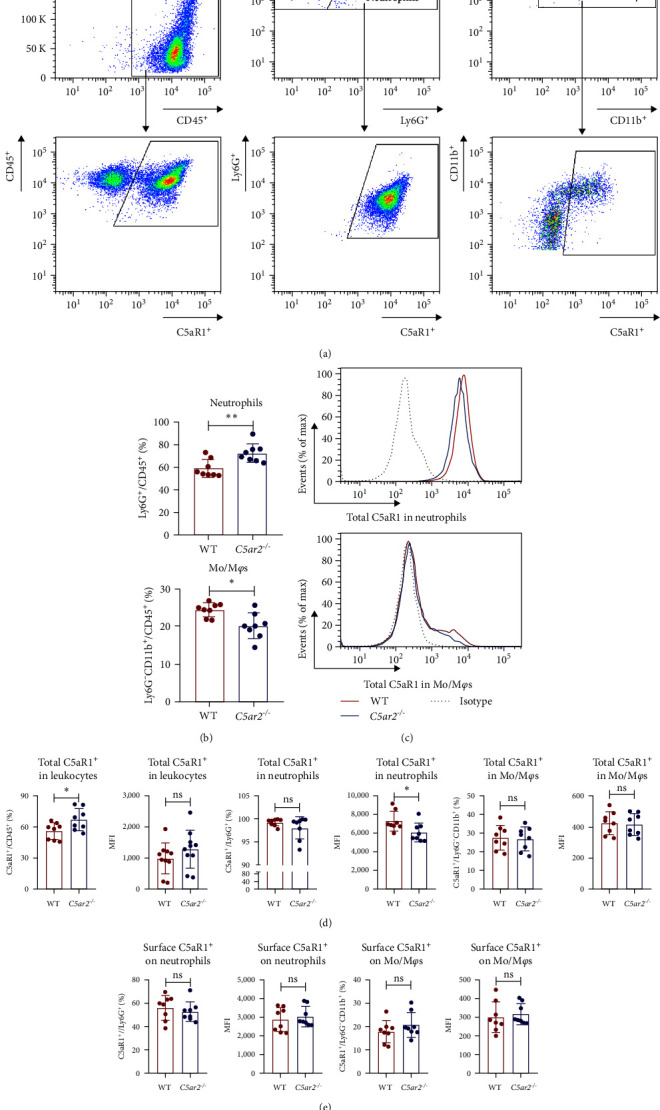
Neutrophil infiltration is higher after 10 hr of 3% TG injection. (a) The representative diagram of peritoneal cells constituent and C5aR1 expression after 10 hr of TG injection. (b) Neutrophils and Mo/M*φ*s infiltration of WT and *C5ar2*^*-/*-^ mice. (c) Representative FACS analysis of total C5aR1 expression in neutrophils and Mo/M*φ*s. (d and e) C5aR1 expression in CD45^+^ leukocytes, in/on neutrophils and Mo/M*φ*s from WT and *C5ar2*^*-/*-^ mice as assessed by flow cytometry (*n* = 8/group). Each dot represents an individual mouse.  ^*∗*^*P* < 0.05,  ^*∗∗*^*P* < 0.01, ns, no significance, Student's *t* test.

In order to find out the C5aR1 levels in neutrophils and Mo/M*φ*s of *C5ar2*^*-/*-^ mice under inflammatory conditions, we harvested mouse peritoneal cells at 10 hr or on 3 days after *i.p*. injection of 3% TG. TG can induce a model of acute peritonitis with a surge increase in peritoneal inflammatory cells. We accessed the constituent of peritoneal inflammatory cells in WT and *C5ar2*^*-/*-^ peritoneal eluates by FACS. After 10 hr of 3% TG injection, there were about 58.9% ± 7.9% neutrophils and 24.2 ± 19.9% Mo/M*φ*s in WT peritoneal leukocytes. Intriguingly, the peritoneal infiltrating neutrophils were higher, while Mo/M*φ*s were lower in *C5ar2*^*-/*-^mice compared with those of WT counterparts (Figures 4(a) and 4(b)). There were no differences in the percentages of total C5aR1^+^ and surface C5aR1^+^ in the peritoneal infiltrating neutrophils and Mo/M*φ*s between these two mouse strains (Figures 4(c), 4(d), and 4(e)). The MFI of C5aR1 in infiltrating neutrophils was slightly lower in *C5ar2*^*-/*-^ mice, with significance (Figures 4(c) and 4(d)). However, the percentage of the total C5aR1^+^ in infiltrating *C5ar2*^*-/*-^ leukocytes was higher than those in WT ones. It is likely due to the increased infiltrating neutrophils in *C5ar2*^*-/*-^mice.

### 3.5. C5aR2 Deficiency Lessens C5aR1 Distribution and Expression in M*φ*s after 3 Days of 3% TG Injection

**Figure 5:**
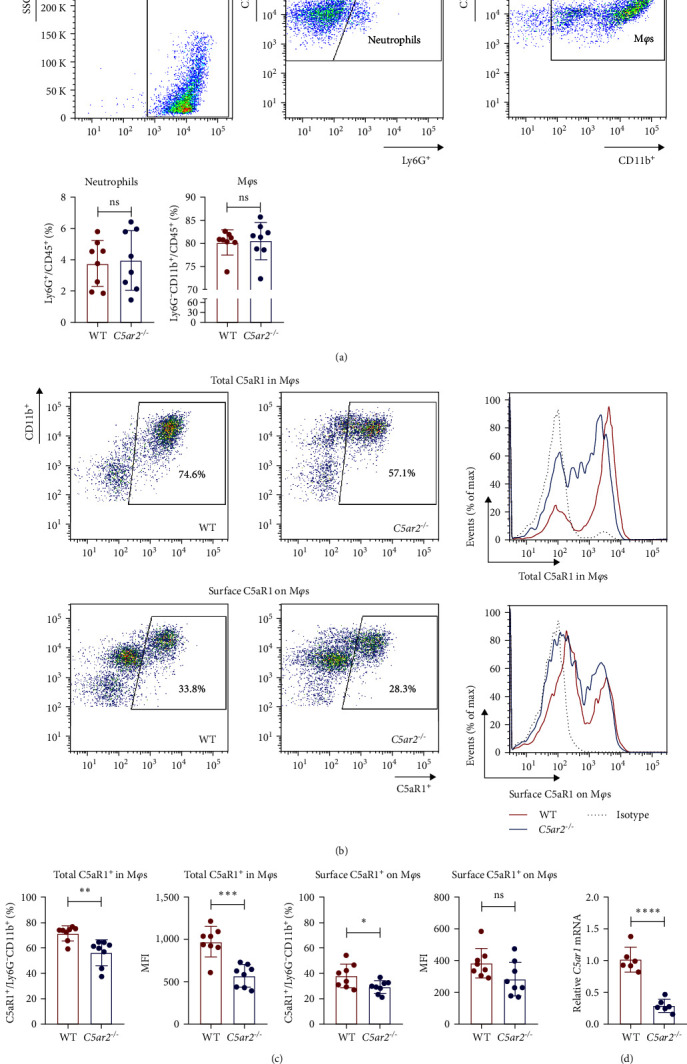
C5aR2 deficiency lessens C5aR1 distribution and expression in M*φ*s after 3 days of 3% TG injection. (a) The representative diagram and statistic graph of peritoneal cells constituent between WT and *C5ar2*^*-/*-^ mice after 3 days of TG injection (*n* = 8/group). (b) Representative FACS analysis of surface and total C5aR1 expression in M*φ*s. (c) Surface and total C5aR1 expression in M*φ*s from WT and *C5ar2*^*-/*-^ mice as assessed by flow cytometry (*n* = 8/group). (d) Relative mRNA levels of C5aR1 between WT and *C5ar2*^*-/*-^ peritoneal cells 3 days after TG injection determined by RT-qPCR. Each dot represents an individual mouse (*n* = 6/group).  ^*∗*^*P*  < 0.05,  ^*∗∗*^*P*  < 0.01,  ^*∗∗∗*^*P*  < 0.001,  ^*∗∗∗∗*^*P*  < 0.0001, ns, no significance, Student's *t* test.

Neutrophils have a short lifespan in circulation and tissue. After the *i.p*. TG injection, neutrophils were recruited to mouse abdominal cavity, peaked, and then died off quickly. Till 3 days, the average neutrophils in peritoneal cells were about 3.8% ± 1.5% and 4.0% ± 1.9% in WT and *C5ar2*^*-/*-^ mice, respectively (Figure 5(a)). M*φ*s were the major cells in mouse abdominal cavity after 3 days of 3% TG injection, about 80.2% ± 2.7% and 80.5% ± 4.0% in WT and *C5ar2*^*-/*-^ mice, respectively (Figure 5(a)). Under inflammatory conditions, the FACS results showed that the C5aR1^+^/Ly6G^−^CD11b^+^% and C5aR1 MFI in M*φ*s, the C5aR1^+^/Ly6G^−^CD11b^+^% on M*φ*s were less than those of WT M*φ*s, which suggested that C5aR2 deficiency may lessen C5aR1 distribution and expression in M*φ*s (Figures 5(b) and 5(c)). The lower total C5aR1 in *C5ar2*^*-/*-^ peritoneal cells was supported by RT-qPCR, verifying that the downregulation of C5aR1 in *C5ar2*^*-/*-^ inflammatory M*φ*s was of transcriptional level (Figure 5(d)).

### 3.6. C5aR2 Deficiency May Be in Favour of C5aR1 Internalization on Neutrophils upon C5a Stimulation

**Figure 6:**
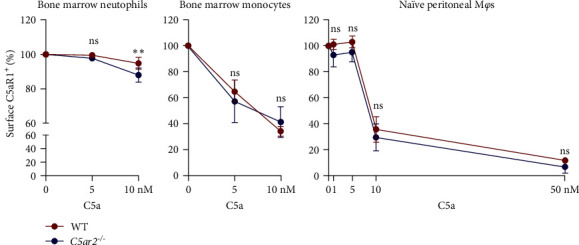
C5aR2 deficiency may be in favor of C5aR1 internalization on neutrophils upon C5a stimulation. Flow cytometry analysis of surface C5aR1^+^% on WT and *C5ar2*^*-/*-^ bone marrow neutrophils, monocytes, and naïve peritoneal M*φ*s after C5a (1–50 nM) stimulation for 30 min. (*n* = 3/group). The surface C5aR1^+^% on these cells with no C5a stimulation was considered as 100%. Each dot represents an individual mouse.  ^*∗∗*^*P* < 0.01, ns, no significance, two-way ANOVA.

Trying to find the reason why surface C5aR1 was lower on *C5ar2*^*-/*-^ naïve circulating neutrophils and inflammatory macrophages, we assessed the internalization of C5aR1 on bone marrow neutrophils, monocytes, and naïve peritoneal M*φ*s. It showed that surface C5aR1^+^% was lower on *C5ar2*^*-/*-^ bone marrow neutrophils after 10 nM C5a stimulation for 30 min compared to WT ones (Figure 6). But there were no significant differences in the internalization of surface C5aR1 on bone marrow monocytes and naïve peritoneal M*φ*s between WT and *C5ar2*^*-/*-^ mice (Figure 6).

## 4. Discussion

The anaphylatoxin C3a and C5a are generally considered as pro-inflammatory mediators. But emerging evidences show the anti-inflammatory role of C3a in certain pathological conditions. Similarly, the functions of C5a are also complex, especially through its receptor C5aR2. Instead of pro-inflammatory factors, it is better and more precise to consider them as inflammatory modulators [20, 21]. C3a binds to its canonical receptor C3aR to exert its inflammatory modulatory functions in health and disease. There are two receptors for C5a, the canonical one C5aR1 and the controversial one C5aR2. Phylogenetic analysis indicates that C5a is the paralog of C3a, it likely evolved from C3a and developed separately in advanced organisms into a chemotactic and spasmogenic molecule [22]. It is interesting to think that during the long process of evolution, it developed another receptor for C5a—C5aR2 to modulate its multi-functions in different internal environments.

Since its discovery, the controversies around C5aR2 continue. But two decades of studies gradually uncover its mysterious veil. C5aR2 localizes on the same chromosome neighboring its paralog C5aR1 and is often concomitantly expressed with C5aR1 [3]. Using a tool mouse -- floxed tandem dye Tomato-C5aR2 knock-in mouse, Karsten et al. [23] tracked the expression of C5aR2 in immune and tissue cells. They found strong and homogeneous C5aR2 expression in neutrophils from bone marrow, blood, and peritoneum, as well as in M*φ*s from peritoneal cavity. But the C5aR2 expression in other tissue M*φ*s is lower and heterogeneous, for example, about 40% of M-CSF-derived bone marrow-derived M*φ*s and 55%–60% of pulmonary M*φ*s are C5aR2^+^. They also found C5aR2 expression in B lymphocytes but not in T lymphocytes.

In our article, we chose neutrophils and peritoneal M*φ*s—two types of cells exhibiting high levels of C5aR2 as the subjects of our study, aimed to investigate the influence of C5aR2 on C5aR1 expression and distribution. As to C5aR1 expression, we found that it is also homogeneous in neutrophils of C57BL/6 mouse, while 5.3% ± 2.7% of WT monocytes from blood, 27.4% ± 6.6% or 71.5% ± 5.8% of WT Mo/M*φ*s from peritoneum 10 h or 3 days after TG injection are C5aR1^+^.

Several animal models have been established to investigate the roles of C5aR2 in different diseases. A significant body of evidence has shown that C5aR2 is far from a recycling decoy receptor, but plays an important role in regulating both pro- and anti-inflammatory responses, depending on different pathological states [19, 24, 25, 26]. To summarize the multi-functions of C5aR2, it is likely that in cells where C5aR2 has concomitant expression with C5aR1, C5aR2 may participate in the signals of C5a-C5aR1 by either promoting or dampening it, C5aR2 may also mediate or crosstalk with certain signals independent of C5a-C5aR1 such as HMGB1 or NLRP3. In tissues or cells where these two receptors are not overlapping, C5aR2 may exert its own set of signals. And recently, a new discovery about C5aR2 function revealed that C5aR2 on endothelium transported C5a generated in the arthritic joint to the blood vessel lumen to initiate C5aR1-driven neutrophil arrest, in a mouse model of immune complex-induced arthritis [27].

The pro-inflammatory roles of C5aR2 and its partner C5aR1—the classical receptor for C5a have been shown in several experimental disease models using *C5ar1*^*-/*-^ and *C5ar2*^*-/*-^ mice, for example, atherosclerosis [28, 29], renal ischemic reperfusion injury [30], and sepsis [11]. Similarly, we have also proved the pro-inflammatory and pathogenic role of C5aR1 and C5aR2 in acute pyelonephritis in mice [18, 19].

Although using the same *C5ar2*^*-/*-^ C57BL/6 mice strain as us, it was reported that C5aR2 deficiency enhanced lung injury and inflammation in a mouse model of immune complex-induced lung injury [7]. Similarly, in another model of LPS-induced lung injury, *C5ar2*^*-/*-^ mice of BALB/c background exhibited enhanced airway edema, more neutrophils as well as pro-inflammatory cytokines and chemokines than WT ones [31]. Intriguingly, in these two models, the neutrophils infiltration significantly increased in bronchoalveolar lavage fluid from *C5ar2*^*-/*-^mice, which was similar to our study in this article of the increased peritoneal neutrophils after 10 hr of TG injection. And these fit with another recent study showing absence of C5aR2 worsened acute tissue injury by increasing C5aR1-mediated neutrophil infiltration in a neutrophil-dependent mouse model of intestinal ischemia-reperfusion [32].

Meanwhile, in mouse models of cecal ligation and puncture-induced sepsis, either C5aR1 or C5aR2 deficiency was protective, reflected in improved survival outcomes or better cardiac functions [11, 33]. Their roles were parallel —pro-inflammatory in peritoneal macrophages and cardiomyocytes. However, it was reported that C5aR2 in blood neutrophils was associated with poor prognosis of sepsis both in humans and rat models [10]. Thus, the functions of C5aR2 may depend on the pathological environments, the functional cells where it is expressed or even its cell location.

In cells where both C5aRs are expressed, the relationship of C5aR2 with C5aR1 seems complex. Surface C5aR2 may antagonize intracellular C5aR1 signals, such as in human CD4^+^ T cells [34]. While intracellular C5aR2 helping with or antagonizing C5aR1 signals were both reported [9, 14, 15, 16]. It may colocalize with C5aR1, or even form heteromer with it. In order to study more about the relationship of these two receptors, we tried to investigate the effects of C5aR2 on C5aR1 expression under the extreme condition of C5aR2 knockout. Our investigation focused on neutrophils and M*φ*s, two cells in which both C5aR1 and C5aR2 are abundantly expressed.

The comparable mRNA levels of C5aR1 in WT and *C5ar2*^*-/*-^ kidney, liver, RTECs, and blood leukocytes from normal mice bred in SPF conditions indicated that despite the proximity of their genes on chromosome 7 in mice, the technology of C5aR2 knockout itself did not measurably affect the transcription of C5aR1 mRNA. This was further verified by unaffected C5aR1 protein expression in bone marrow and circulating neutrophils and monocytes as well as naïve peritoneal M*φ*s, respectively, by FACS detection.

There were conflicts about the surface C5aR1 expression on *C5ar2*^*-/*-^ neutrophils [11, 35, 36]. Our result showed although surface C5aR1 MFI on bone marrow *C5ar2*^*-/*-^ neutrophils seemed lower than that on WT ones, there was no significance, in line with Seiler et al.'s [36] report.

Intriguingly, although surface C5aR1 showed no difference between bone marrow neutrophils, C5aR2 deficiency may affect the distribution of C5aR1 on circulating neutrophil surface, reflected by the reduced surface C5aR1 MFI on *C5ar2*^*-/*-^ neutrophils in peripheral blood. C5aR1 can undergo rapid ligand-dependent internalization [37]. And it was reported that C5aR2 underwent constitutive internalization for the lysosomal degradation of C5 a/C5a des Arg [6]. Would it be possible that the internalization of C5aR1 could be more pronounced upon C5aR2 depletion? Thus, we assessed the effect of C5aR2 deficiency on the internalization of surface C5aR2 on neutrophils and Mo/M*φ*s. There were no significant differences in the internalization of surface C5aR1 on WT and *C5ar2*^*-/*-^ Mo/M*φ*s, which is different from Hsu et al.'s [16]report for the requirement of C5aR2 for C5aR1 internalization. While after 30 min of C5a stimulation (5, 10 nM), surface C5aR1% on *C5ar2*^*-/*-^ bone marrow neutrophils was lower than that on WT ones under high concentration of C5a stimulation (10 nM). The circulation C5a level was very low, only about 0.5 nM [38]. Thus, circulation C5a may not change the surface C5aR1^+^% on WT and *C5ar2*^*-/*-^ neutrophils, but C5a might contribute to the slight downregulation of surface C5aR1 MFI on *C5ar2*^*-/*-^ neutrophils when they moved from bone marrow to circulation due to the ligand-dependent internalization of C5aR1. Chen et al. [35] also suggested the lower C5aR1 on neutrophils could be one way of C5aR2 in optimizing C5a signals, and the upregulation of surface C5aR1 on naïve WT neutrophils by C5aR2 might be one regulating way of GPCR heterodimerization.

However, surface C5aR1^+^% and C5aR1 MFI were not lower on *C5ar2*^*-/*-^ neutrophils at 10 hr after TG injection. The increased internalization of C5aR1 on *C5ar2*^*-/*-^ neutrophils might be concealed by the increased number of infiltrating neutrophils. It is reported that although intestinal IR in *C5ar2*^*-/*-^ mice led to worsened intestinal damage and increased neutrophil infiltration, the intestinal pro-inflammatory cytokines were reduced [33]. We have also found reduced pro-inflammatory cytokine IL-1*β* from *C5ar2*^*-/*-^ peritoneal cells after 1 day of TG injection, upon LPS stimulation [19]. Neutrophils were about 30% in 1 days' peritoneal cells, and there was no difference between WT and *C5ar2*^*-/*-^ mice (data not shown). So the reduction of total C5aR1 MFI in *C5ar2*^*-/*-^ neutrophils from peritoneal cavity after TG injection might contribute to the reduced inflammation amplified by LPS.

There were no differences in peritoneal infiltrating neutrophils, M*φ*s between WT and *C5ar2*^*-/*-^ mice after 3 days of TG injection. M*φ*s made up the majority of these cells, which accounted for about 80%. Although C5aR1 expression was not affected in naïve M*φ*s, we found that surface and total C5aR1 expression in inflammatory M*φ*s was lower in *C5ar2*^*-/*-^ mice compared to WT mice. This downregulation was of mRNA level. The model of peritonitis triggered acute inflammatory responses which would include the secretion of a plethora of inflammatory mediators including the activation of C5-producing C5a. C5a would bind and activate C5aR1 leading to the internalization of C5aR1 on immune cells. But unlike neutrophils, we could not lead to the speculation that the lower surface C5aR1 on inflammatory M*φ*s might be due to the internalization of C5aR1 since we did not find the influence of C5aR1 internalization on naïve *C5ar2*^*-/*-^ Mo/M*φ*s. It might be a reflection of the reduced total C5aR1 pool in *C5ar2*^*-/*-^ M*φ*s. In our previous study, we have already found reduced IL-1*β*, CXCL-1, and TNF*α* in *C5ar2*^*-/*-^ M*φ*s harvested on 3 days post TG injection, compared with WT littermates, upon LPS stimulation [19]. It is uncertain whether this downregulation of C5aR1 was of direct modulation of C5aR2 signal or the indirect influence. However, C5aR2 deficiency and the reduction of total C5aR1 in *C5ar2*^*-/*-^ macrophages from peritoneal cavity 3 days after TG injection may account for the reduced inflammation. Thus, in the inflammatory state of peritonitis, C5aR1 and C5aR2 signals might cooperate in modulating macrophage inflammatory responses.

## 5. Conclusions

Collectively, although C5aR2 deficiency lessens C5aR1 distribution and expression in neutrophils and M*φ*s, C5aR2 may function differently between them. In neutrophils, C5aR2 may antagonize the internalization of C5aR1 and the chemotaxis signal upon C5a and C5aR1 interaction, reflected in the slight downregulation of C5aR1 MFI on circulating *C5ar2*^*-/*-^ neutrophils and more infiltrating neutrophils in peritoneal cavity of *C5ar2*^*-/*-^ mice at 10 hr after TG injection. In M*φ*s, C5aR2 may cooperate with C5aR1 signal in the secretion of inflammatory factors, as C5aR2 deficiency reduced surface and total C5aR1 in M*φ*s in inflammatory peritoneal environment.

C5aR1 can couple to both Gi and *β*-arrestins upon activation by C5a, while C5aR2 solely signals via *β*-arrestins without G-protein activation [2]. The pressing question is the precise mechanism of why the quaternary of C5aR1, C5aR2, G-proteins, and *β*-arrestins functions biasedly in cells or under circumstances such as different C5a concentrations. Recently, the mechanism of activation and biased signaling in C5aR1 using cryo-electron microscopy was reported [2, 39]. It is encouraging that the gap in C5aR2 research will be filled before long.

## Figures and Tables

**Table 1:** PCR primers.

Primer ^*∗*^	Sequence	Product size (*bp*)	Accession number
*β*-Actin-1	5′-CACACCCGCCACCAGTTCG-3′	70	NM_007393.5
*β*-Actin-2	5′-ACATGCCGGAGCCGTTGTC-3′		
Gapdh-1	5′-ACCACAGTCCATGCCATCAC-3′	452	NM_001411843.1
Gapdh-2	5′-TCCACCACCCTGTTGCTGTA-3′		
C5ar1-1	5′-CAGGACATGGACCCCATAGAT-3′	155	NM_007577.2
C5ar1-2	5′-ACCAGGAACACCACCGAGTAG-3′		

^*∗*^ Primer-1 is forward primer; primer-2 is reverse primer.

## Data Availability

Data will be available upon reasonable request.

## Abbreviations

C5aR1: Complement 5a receptor 1
C5aR2: Complement 5a receptor 2
GPCR: G-protein-coupled receptor
*i.p*.: Intraperitoneal
MFI: Mean fluorescence intensity
Mo/M*φ*s: Monocytes/macrophages
RT-PCR: Reverse transcriptional-polymerase chain reaction
RTECs: Renal tubular epithelial cells
TG: Thioglycollate.

## Supplementary Materials

Supplementary Materials Figure S1: the verification of *C5ar2*^*-/*-^ mice in gene and protein level. Figure S2: the *C5aR2*^*-/*-^ peripheral blood leukocytes without any fluorescence antibody staining have green fluorescence where C5aR2 should express. Figure S3: the stepwise gating strategy used in flow cytometric analysis of peripheral blood leukocytes, neutrophils, monocytes and their total C5aR1expressionaccordingto the isotype. Figure S4: C5aR1 distribution and expression are similar in naïve bone marrow neutrophils and monocytes between WT and *C5ar2*^*-/*-^ mice. Figure S5: there are no differences of total C5aR1 expression in peripheral blood neutrophils and monocytes from WT and *C5ar2*^*-/*-^ mice in genders. *n* = 5–8/group. Each dot represents an individual mouse. Two-way ANOVA.
