# Supplementary material for: C5aR2 Deficiency Lessens C5aR1 Distribution and Expression in Neutrophils and Macrophages
Source: J Immunol Res. 2024 Jul 10;2024:2899154. doi: 10.1155/2024/2899154 (PMC11254461; doi:10.1155/2024/2899154)

C5aR2 deficiency lessens C5aR1 distribution and expression in neutrophils and macrophages

Figure S1: The verification of *C5ar2*$^{-/-}$ mice in gene and protein level. (A) Genetyping of *C5aR2*$^{-/-}$ mice. PCR analysis of tail genomic DNA from WT and *C5ar2*$^{-/-}$ mice of one breeding pair respectively using two pairs of primers for *C5ar2* and *Neomycin*. The agarose gel shows positive *C5ar2* in WT mice, negative *C5ar2* but positive *Neomycin* in *C5ar2*$^{-/-}$ mice, confirming the gene knockout of *C5ar2* and the introducing of *Neomycin* during this process. The 100-bp DNA markers (M) are shown alongside the gels. (B) Representative FACS analysis of C5aR2 expression in peritoneal Mφs cells 3 days after TG injection.

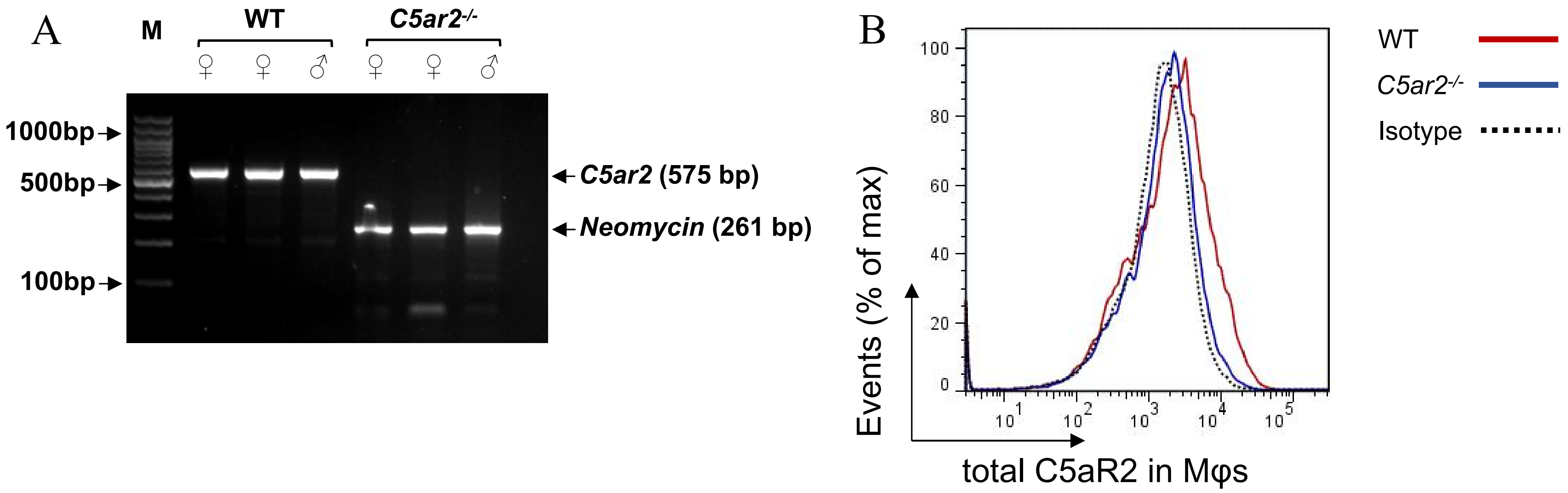

Figure S2: The *C5aR2*$^{-/-}$ peripheral blood leukocytes without any fluorescence antibody staining have green fluorescence where C5aR2 should express.

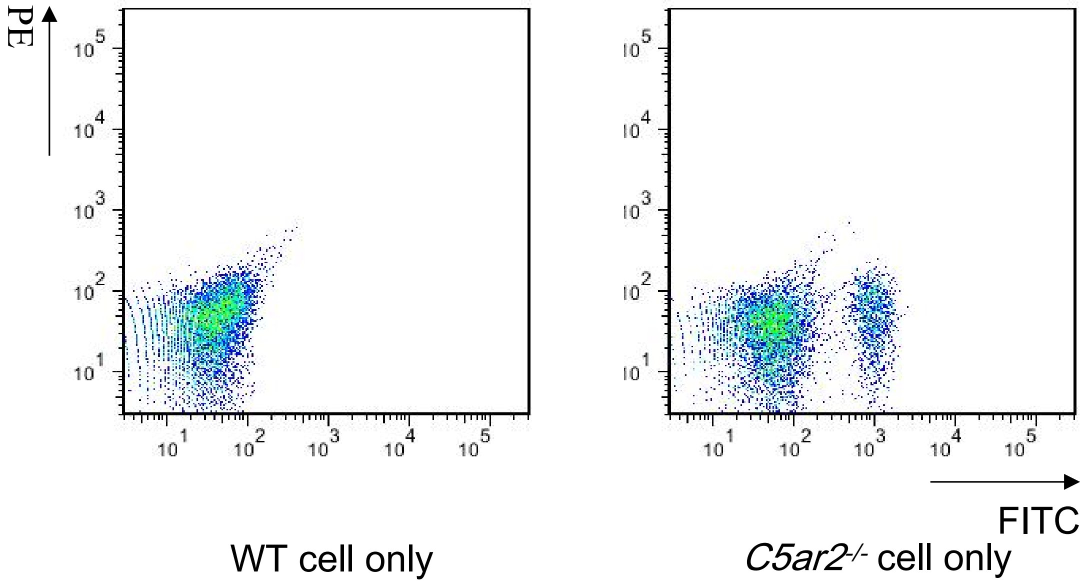

Figure S3: The stepwise gating strategy used in flow cytometric analysis of peripheral blood leukocytes, neutrophils, monocytes and their total C5aR1 expression according to the isotype.

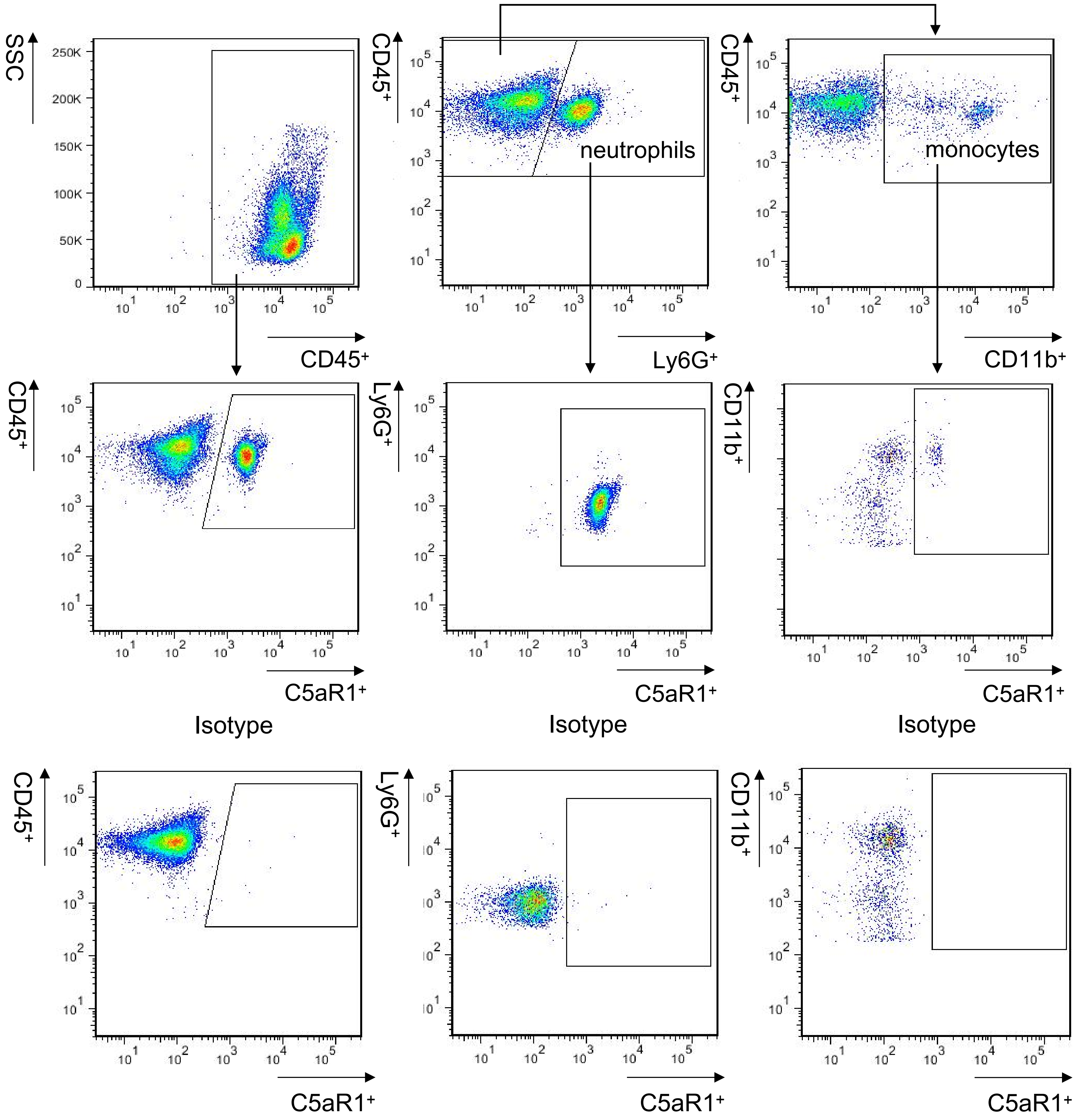

Figure S4: C5aR1 distribution and expression are similar in naïve bone marrow neutrophils and monocytes between WT and *C5ar2*$^{-/-}$ mice. (A) The percentages of neutrophils and monocytes in bone marrow $CD45^+$ leukocytes from normal WT and *C5ar2*$^{-/-}$ mice as assessed by flow cytometry. (B) Surface and total C5aR1 expression in naïve bone marrow neutrophils and monocytes from WT and *C5ar2*$^{-/-}$ mice as assessed by flow cytometry. n = 8/group. Each dot represents an individual mouse. ns, non significance, Student's *t* test.

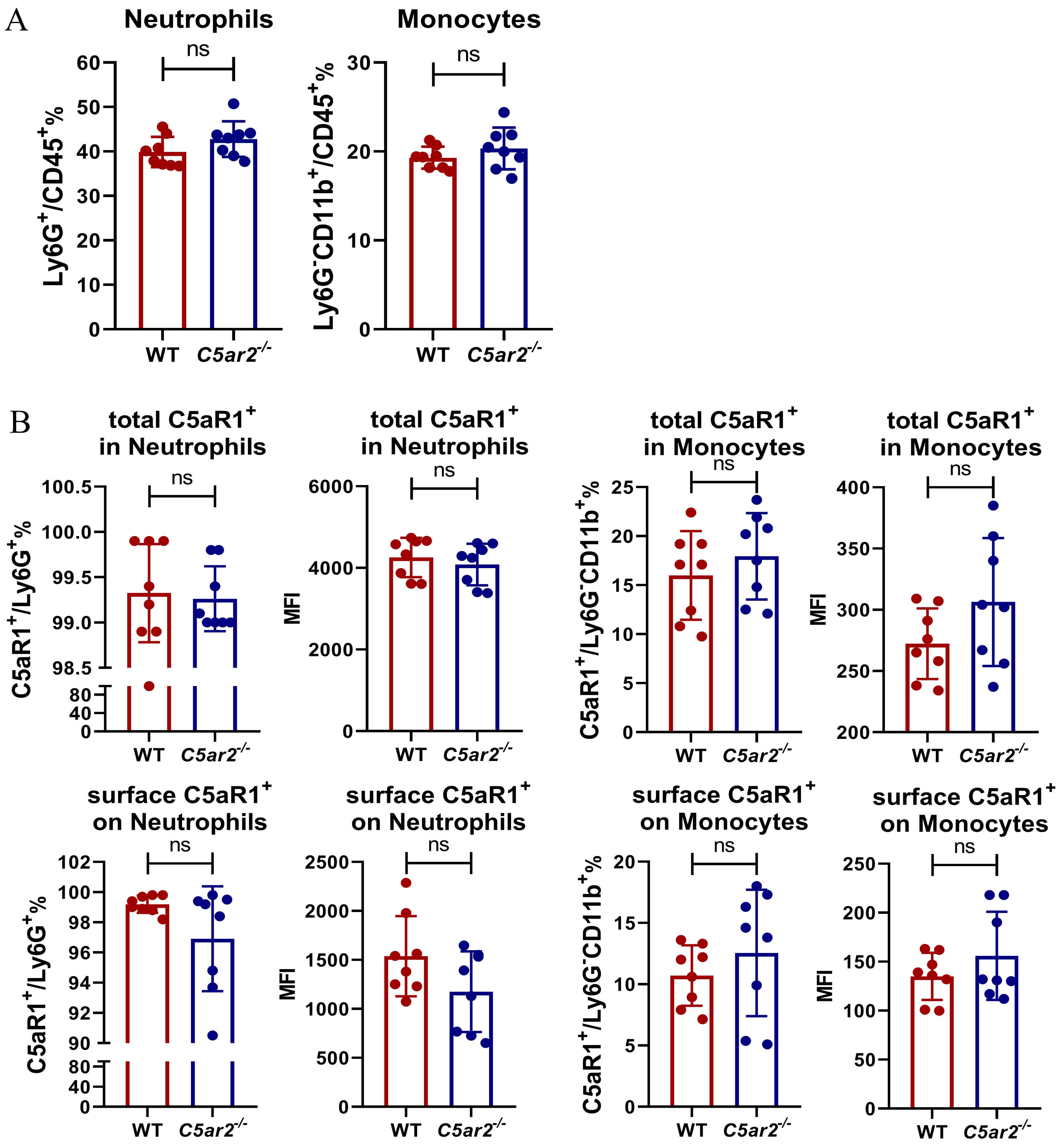

Figure S5: There are no differences of total C5aR1 expression in peripheral blood neutrophils and monocytes from WT and *C5ar2*$^{-/-}$ mice in genders. n = 5-8/group. Each dot represents an individual mouse. Two-way ANOVA.

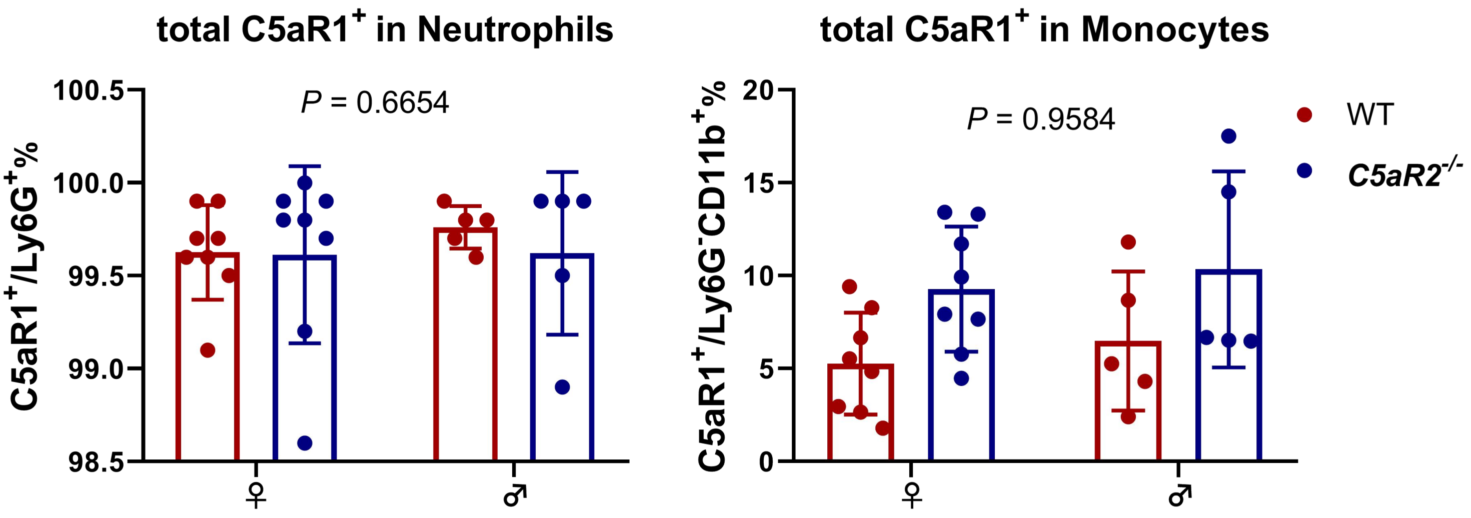

Supplement: Supplementary Materials — Figure S1: the verification of C5ar2-/- mice in gene and protein level. Figure S2: the C5aR2-/- peripheral blood leukocytes without any fluorescence antibody staining have green fluorescence where C5aR2 should express. Figure S3: the stepwise gating strategy used in flow cytometric analysis of peripheral blood leukocytes, neutrophils, monocytes and their total C5aR1expressionaccordingto the isotype. Figure S4: C5aR1 distribution and expression are similar in naïve bone marrow neutrophils and monocytes between WT and C5ar2-/- mice. Figure S5: there are no differences of total C5aR1 expression in peripheral blood neutrophils and monocytes from WT and C5ar2-/- mice in genders. n = 5–8/group. Each dot represents an individual mouse. Two-way ANOVA. [file 2899154.f1.pdf]
